# Interplay of Regulatory T Cell and Th17 Cells during Infectious Diseases in Humans and Animals

**DOI:** 10.3389/fimmu.2017.00341

**Published:** 2017-04-03

**Authors:** Sharvan Sehrawat, Barry T. Rouse

**Affiliations:** ^1^Indian Institute of Science Education and Research Mohali, Mohali, Punjab, India; ^2^Department of Biomedical and Diagnostic Sciences, College of Veterinary Medicine, The University of Tennessee, Knoxville, TN, USA

**Keywords:** T regulatory cells, Th17 cells, cross-regulation, humans, animals, outbred population

## Abstract

It is now clear that the outcome of an inflammatory process caused by infections depends on the balance of responses by several components of the immune system. Of particular relevance is the interplay between regulatory T cells (Tregs) and CD4^+^ T cells that produce IL-17 (Th17 cells) during immunoinflammatory events. In addition to discussing studies done in mice to highlight some unresolved issues in the biology of these cells, we emphasize the need to include outbred animals and humans in analyses. Achieving a balance between Treg and Th17 cells responses represents a powerful approach to control events during immunity and immunopathology.

## Introduction

**Figure 1:**
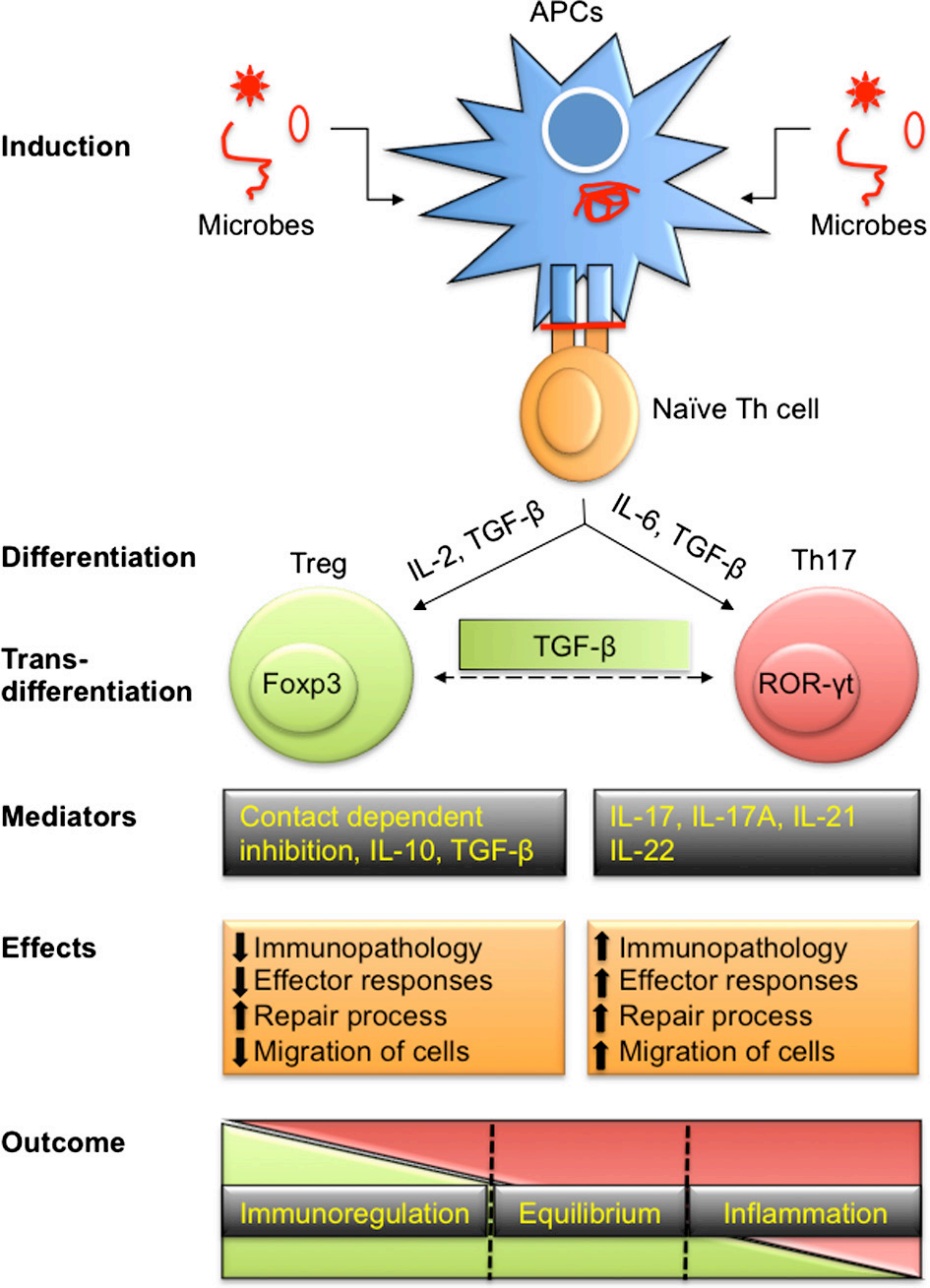
**Differentiation of T regulatory cells (Tregs) and Th17 to effect immunity and immunopathology during infections**. Antigen-presenting cells (APCs) either by direct infection or by exogenously taking up antigens process polypeptides intracellularly to generate peptides. These peptides are loaded onto class II MHC molecules and presented on their surface to activate naïve Th cells. Depending upon the affinity of TCR to recognize processed peptides and the microenvironment in which such interactions take place, Th cells are polarized into pTreg and Th17 cells to maintain homeostasis. Treg and Th17 cells can also transdifferentiate depending on intrinsic as well as some extrinsic factors such as local concentration of TGF-β. Predominant products of Treg include IL-10, IL-35, and TGF-β in addition to membrane-expressed molecules while Th17 cells secrete IL-17, IL-21, IL-22, and other cytokines. Tregs cause immunoregulation while Th17 serve as proinflammatory cells during disease progression. Treg and Th17 leads to differential outcome ranging from dominant regulatory to stimulatory activity while a fine balance ensures homeostasis.

The realization that CD4^+^ T cells could be differentiated in two phenotypically separate lineages, Th1 cells that predominantly produce IFN-γ and IL-2 while Th2 cells produce IL-4 and IL-10, was elucidated by Mosmann et al. (1). The idea caught on because these cell types cross-regulated each other and this phenomenon helped in explaining many observations in inflammatory and infectious diseases. Subsequently, several additional subtypes of CD4^+^ T cells were discovered based on the transcription factor expressed, their cytokine profile and functions (2, 3). Of particular relevance was the discovery that some CD4^+^ T cells play a regulatory role and helped to constrain the effector function of other cell types. We currently recognize at least four CD4^+^ T cell subsets which largely play an effector function (Th1, Th2, Th9, and Th17) and another subset T follicular helper cell (T_FH_) which plays a major role during immune induction (4). This review focuses largely on the cross play between regulatory T cells (Tregs) and Th17 cells since these two subsets often subserve opposite roles during inflammatory processes. Th17 cells are recognized as one of the predominant proinflammatory cell types and produce IL-17 to help attract other innate immune cells such as macrophages and neutrophils to further aggravate chronic inflammation. The transcription factor RAR-related orphan receptor (ROR)-γt regulates the speciation program of Th17 cells. Tregs on the other hand act to regulate the differentiation and activity of Th17 cells. In fact, several lines of evidence demonstrate that Treg and Th17 cells exhibit some key shared differentiation pathways (Figure 1). Thus, both cell types require TGF-β and IL-2 for their differentiation and are predominantly present in the gut to maintain homeostasis (5). Both Treg and Th17 cells exhibit specificity toward commensal-derived antigens or self-antigens and their speciation transcriptional program shows direct interaction (5). Of the two major classes of antigen-presenting cells (APCs) in the gut, dendritic cells (DCs) are known to promote Th17 cell responses while macrophages promote Treg responses (6). Treg and Th17 cells were shown to predominantly maintain gut homeostasis but their interplay in other diseases that include those caused by infections is beginning to be appreciated.

The idea that T cells could suppress the function of other cells was popularized by Gershon and Kondo (7). The cells were called suppressor cells, but since there were no reliable means of identifying them, they soon fell into disrepute. Resurrection and respectability for Treg came some two decades later by the work performed by the Sakaguchi et al. and Suri-Payer et al. who had discovered a reliable way of distinguishing Treg from other cell types and also demonstrated their regulatory effects (8–10). Sakaguchi et al. and Thornton and Shevach demonstrated that 5–10% of T helper (Th) cells that expressed the high affinity IL-2 receptor alpha (α) chain (CD25) were present in naïve mice and were able to suppress the proliferation of those cells that did not similarly express this molecule (9, 11). The idea of Treg’s existence helped in explaining many unsolved mysteries in immunobiology such as how tolerance is maintained and the variable outcome of autoimmune and infectious diseases is effected (12–14). The canonical transcription factor Fork head box protein 3 (Foxp3), responsible for controlling the function of Treg and acting as their identifier, was discovered in 2001 (15–17). Whereas Foxp3^+^ Tregs are perhaps the most prominent regulatory cells, other cell types have been observed to mediate regulatory effects alongside or alternatively to Foxp3^+^ Treg. These many alternative regulators include Tr1 cells, Th3 cells, CD8^+^ Treg, double negative CD3^+^ T cells, gamma delta (γδ) T cells, natural killer T cells, regulatory B cells, myeloid-derived suppressor cells, and perhaps others.

A great majority of our understanding of how the immune system works comes from studies performed in inbred mice housed in controlled environment. Our ultimate objective, however, is to understand the workings of the immune system and to apply the wisdom to manipulate the outcome of events in humans and other animals. There is still a gap in our knowledge regarding what happens in humans and outbred non-rodents and this issue is elaborated in this review. We also discuss unresolved issues in the biology as well as pathophysiology of Treg and Th17 cells during infectious diseases.

## Biology of Treg and Th17 Cells

The expression of the transcription factors Foxp3 and ROR-γt defines Treg and Th17 cells, respectively. Foxp3 is critically involved in the differentiation and function of Treg. Foxp3 does so by directly binding to DNA to be transcribed and in so doing regulates the transcription of more than thousand genes many of which are involved in T cell activation. Some of the Treg-specific genes directly targeted by Foxp3 are *Il2ra* (CD25), *Tnfrsf18* (GITR), *Nrp1* (neuropillin-1), and *Ccr4* among others (18–20). Foxp3 could also influence gene expression indirectly by recruiting epigenetic modifiers such as histone deacetylases (HDAC1, 2, and 3) in the complex (21). Many genes that include *Il2* are downregulated by HDACs activity. As newer mechanistic insights are emerging, clearly there is need of more studies to better define the role of Foxp3 in programming Treg and in fact different functions could be attributed to its different domains. Similarly, Foxp3 regulates the expression of some chemokine receptors suggesting that it may also control the homing of Treg. The latter effect has not received much attention and needs to be understood in greater detail. This is because immunosuppression at inflammatory sites is one of the most desirable outcomes of cell-based immunotherapies.

Tregs are broadly divided into thymically derived regulatory T cells (tTregs) and those that are induced in the periphery (pTregs). pTregs are usually more plastic than tTregs (22). Nrp1 may act as the distinguishing marker between tTreg (+) and pTreg (−) (23–25). Tregs in the thymus develop after 3 days of birth and a thymectomy at 3 days of birth abrogates Treg responses leading to multiorgan autoimmune inflammatory diseases (26). However, some Treg that specifically home to select lymphoid organs can be detected in 3-day-old thymectomized mice (27). Therefore, it could be that the kinetics of Treg generation in the thymus is also linked to their differential homing pattern. As and when growing animals are exposed to different environmental conditions that include feed and habitation, the homing properties, functionality, and repertoire of Treg may be refined further to maintain homeostasis at different locations.

For the induction of T cell responses that include Treg, three signals comprising MHC–peptide–TCR, engagement of co-stimulatory/inhibitory molecule, and cytokines in milieu are required (28, 29). Issues such as the strength and the nature of inducing signals and the subsequent formation of either plastic or stable Treg are beginning to be investigated (30). Low to intermediate affinity interactions between the TCR expressed by developing T cells and peptides–MHC class II complexes in thymus are considered as one of the critical drivers of Treg differentiation (28). Contrary to what was considered as a paradigm that both α and β chains of the TCR are involved in peptide binding (31), a recent study demonstrated that only the β chain of TCR along with its framework regions contributed to peptide binding in Tr1 cells and thereby making it a very low affinity interaction (32, 33). However, one wonders how such a weakly interacting TCR ensures survivability of T cells during the thymic selection process. Whether or not TCRs of different types of Treg also display a similar orientation and affinity remains unexplored.

The affinity with which TCRs of Th17 cells recognize peptides has not been extensively explored. Only a few studies have demonstrated that TCRs of Th17 cells might exhibit a low affinity (34). High affinity interactions in fact might be counterproductive for gut health, a site so heavily infested by microbes. Thus, in healthy individuals a unique tripartite interaction among gut microbiota, Treg, and Th17 cells may be required to maintain gut homeostasis (35). Conceivably, Th17 cells act to control the excessive growth of microbes in the gut while Tregs regulate Th17 cell responses. Whether Th17 cells exhibit differential TCR specificity or affinity toward antigens and how it affects their pathogenicity is worth investigating and could indeed help identify Th17 cell subsets with different functions. Some studies have supported a similar idea that Th17 cells could indeed exist in different subtypes (36–38). Accordingly, a local intracellular concentration of saturated fatty acids (SFA) compared to polyunsaturated fatty acids (PUFA) favored more pathogenic Th17 cell formation (38). Differential accumulation of SFA or PUFA and their binding to intracellularly expressed CD5L led to the generation of Th17 exhibiting differential pathogenicity (38).

The stimulating antigens for Treg and perhaps for Th17 cells could also be generated during an ongoing inflammatory response caused by autoimmune diseases or infections. To support this notion, a few studies have demonstrated that Tregs isolated from draining LNs are more active and better suppressors as compared to those isolated from distal LNs (39–42). In draining LNs, APCs home from local sites and predominantly sample antigens released from these areas. This provides ample stimulation for Treg to remain better suppressors.

TGF-β is a critical cytokine required at least *in vitro* for inducing the regulatory phenotype in T cells. Depending on the concentration, context, and condition, TGF-β helps skew responses toward Treg or Th17 cells (43, 44). Thus, a greater concentration of TGF-β may be conducive for a Treg response while a lower concentration particularly in the presence of other inflammatory cytokines such as IL-6 and IL-21 could preferentially promote Th17 responses (45). In fact, some pathogens either encode for the homologs of TGF-β or help activate latent TGF-β and this may be responsible for differential proinflammatory or regulatory responses (46). Whether or not TGF-β is critical for Treg generation in the thymic environment was investigated in the absence of TGF-β signaling using complete knockout or T cell specific TGFβRII knock out mice (47–49). These studies revealed that an absence of TGF-β signaling only affected the peripheral pool of Treg and not their thymic generation (47). It could also suggest that Treg that develop in the thymus halt their proliferation and remain quiescent until they home to the periphery. The reduced proliferation of Treg in the thymus could be the consequence of limited antigen availability and the presence of abundant TGF-β, both of which could serve to induce slow proliferation of Treg (48). The thymic microenvironment could indeed provide copious amount of TGF-β for Treg differentiation or maintenance because of an ongoing process of apoptosis and disposal of such cells by phagocytic activity of DCs and macrophages (49). Another signal that has been implicated in Treg generation is retinoic acid, a metabolite of vitamin A (50). The expression of TGF-β and retinoic acid has also been demonstrated in the thymus supporting the notion that these induction pathways either alone or cooperatively could help thymic Treg generation (51–53).

IL-2 signaling is critically involved in Treg as well as Th17 cell differentiation. IL-2 is consumed preferentially by Treg since they express high affinity IL-2 receptors (54). IL-2 also acts to stabilize Foxp3 induced by TGF-β (30, 55). Treg are supposed to dampen inflammation where a mix of both pro- and anti-inflammatory cytokines constitutes the microenvironment. Therefore, the functionality of Treg needs to be evaluated in the presence of relative abundance of different cytokines. TGF-β and IL-2, if present in an environment along with other proinflammatory cytokines such as IL-1β, IL-6, IL-21, or IL-23, facilitate Th17 differentiation at the expense of Treg (56). It is worth investigating how ROR-γt in Th17 cells actually promotes their programming. Thus, whether or not the transcription factor ROR-γt in Th17 cells actually binds in the promoter region of IL-17 to modulate its expression has not been shown experimentally. However, a putative binding site of ROR- γt in IL-17 promoter has been predicted (57). Similarly, any naturally existing endogenous ligands for ROR-γt is yet to be identified. The induction kinetics of such ligands during infection could provide better insights into the differentiation of Th17 cells during an ongoing immune response and provide potential targets to block a pathogenic response. One such example is binding of an artificial ligand digoxin to ROR-γt which acts to diminish IL-17 production (58). The factors shown to favor and antagonize Treg and Th17 cells in different species are summarized in Table 1. In a subsequent section, we highlight technological advances that facilitated Treg or Th17 cell response investigations as well as their interplay and also make some comments about their potential therapeutic value.

**Table 1:** **A summary of positive and negative regulators of regulatory T cell (Treg) and Th17 cell response in different species**.

Species	Tregs		CD4^+^ T cells that produce IL-17	
	Promoting factors	Inhibiting factors	Promoting factors	Inhibiting factors
Mouse	IL-2	IL-6	IL-6	TGF-β (high concentration)
	TGF-β	TNF-α	TGF-β (low concentration)	IL-2
	IL-10	IL-1β	IL-21	IL-4
	Lower affinity of TCR		IL-23	IL-12
			Saturated fatty acids	IFN-γ
			IL-1β	Polyunsaturated fatty acids
				Estradiol

Human	Antigen or mitogen	IL-6	IL-6	TGF-β
	IL-2	IL-21	TGF-β (low concentrated)	IL-4
	TGF-β	IL-23	IL-21	IL-12
		IL-17	IL-23	IFN-γ
		TNF-α		
		IL-1β		
		RANTES		

Canine (dogs)	Con-A	IL-6	IL-6	TGF-β
	IL-2	IL-1β	IL-1β	
	TGF-β		TFG-β	
	IL-10			

Feline (cats)	Mitogens	IL-6	IL-1β	TGF-β
	LPS and flagellin	IL-1β	IL-6	IL-10
	IL-2		TGF-β	
			IL-21	

Bovine	Antigen or mitogens along with IL-10, TGF-β	IL-6	IL-23	Progesterone, IFN-γ

## How Do We Study the Function and Phenotype of Treg and Th17 Cells?

**Figure 2:**
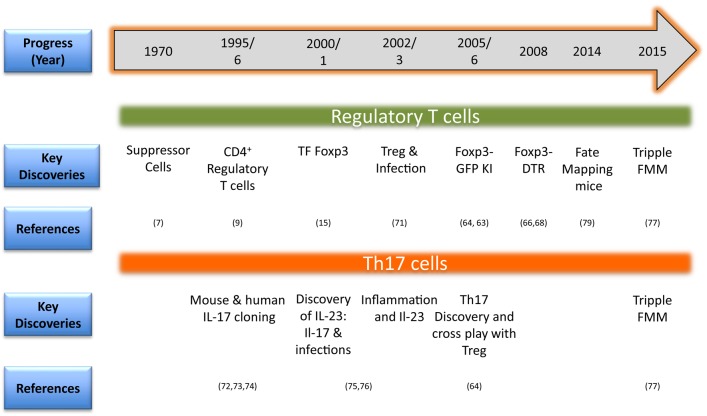
**Timeline in brief for methodological advances for investigating regulatory T cell (Treg) and Th17 responses in animals (TF, transcription factor; KI, knock-in-mice; DTR, diphtheria toxin receptor; FMM, fate mapping mice) (71–76)**.

A summary of the key technological advancements that has facilitated studies involving phenotype and function of Treg and Th17 cells is provided in Figure 2. One of the initial identifiers of Treg in naïve mice was surface expression of CD25 (IL-2R α chain) and this served as a marker to facilitate their isolation and characterization (10). The discovery of the *bona fide* transcription factor Foxp3 advanced the field since it is distinctive of Treg and separates them from non-Treg during ongoing infections. Foxp3 not only confers Treg with their regulatory function but also is used for monitoring Treg responses during disease progression to serve as a prognostic biomarker (15, 16, 59–62). As Foxp3 is expressed intracellulary, its detection requires cells to be permeabilized, which renders them dysfunctional, and hence limits utility. The issue was addressed in inbred mice by Bettelli et al. and Fontenot et al. who constructed a Foxp3-GFP knock-in mouse so that live cells could be recovered based on GFP positivity (63, 64). This model also allowed studying migration and localization of Treg during infections (65–67). Other transgenic mouse models such as Foxp3-diphtheria toxin receptor (DTR) also helped advance our understanding of the function and pathophysiology of Treg especially during ongoing infections and immune activation. The DTR is not naturally present in mice and, therefore, a selective depletion of Treg could be achieved by injecting minimal dose of diphtheria toxin (13, 66, 68, 69). Many studies employed this model to study the role of Treg during different stages of an ongoing infection or autoimmune disease (66, 70).

A confounding problem, however, complicated matters since it was realized that Treg might lose their expression of Foxp3 as well as their regulatory function. Moreover, such cells could even take on the function of effector T cells (4). This phenomenon is usually referred to as plasticity or the transdifferentiation. This can be investigated with the availability of so-called fate mapping mice (77, 78). Such animals are constructed in a way that desirable gene products such as Foxp3 or IL-17 are driving Cre recombinase. Crossing these animals with reporter floxed mice having a transgene for fluorescent protein generated fate-mapping mice to address plasticity issues during infections and other inflammatory situations (77, 79). Whether or not proinflammatory cells producing IL-17 could also become regulatory at a later time, triple fate mapping mice have now been created (77). Using these animals, it was demonstrated that Th17 cells could transdifferentiate into Tr1 cells in a model of parasite induced inflammatory disease (77). Thus, fate-mapping mice have become a valuable model to follow the functional changes of T cell subsets in different situations. The method of generation such as inducible vs constitutive expression of transgene/reporter, number of copies inserted, and the expression of products under non-endogenous promoters could, however, impact on the overall utility of such animal models (80). Whether or not Treg plasticity occurs in humans has been difficult to quantify and co-staining for different markers followed by multicolor flow cytometry represents one surrogate way to measure it. In order to generate phenotypically stable regulatory T cells, approaches that modify epigenetic architecture are used. For example, epigenetic modifiers such as HDACs or DNA methyltransferases (DNMTs) inhibitors are used. The use of azacytidine that inhibits DNMTs activity ameliorated herpes simplex virus 1 (HSV-1) induced ocular inflammatory lesion and enhanced Treg responses (81).

In order to gain insights into the functioning of Treg or Teffectors in lymphoid organs or in inflammatory tissues, cells need to be visualized *in vivo*. This could be achieved using two photon intravital microscopy but its accessibility is limited (82, 83). Many observations obtained using inbred strains may not translate to outbred populations for reasons such as the representation of limited MHC polymorphism in former animals. In addition, spontaneous exposures of feral animals to multiple antigens as compared to those that are housed in clean facilities may also yield confounding conclusions. That a dirty environment can make a difference is being emphasized and may include differential migration pattern of immune cells as was shown for CD8^+^ T cells (84, 85). This led to differential outcome during a subsequent viral infection (84). There is no reason to believe that such a situation would not exist for Th subsets and other types of infections.

One model that could be valuable to address such issues is the zebrafish (*Danio rerio*) (86). The model could be particularly valuable to study cellular interactions due to its anatomical visual transparency. For investigating Treg and Th17 responses in zebrafish, the genes encoding for transcription factors Foxp3 and ROR-γt have been cloned successfully (87). The immune cells and molecules known to exist in vertebrates critical for adaptive immunity are also present in zebrafish (88). Procedures such as transgenesis, nuclear reprogramming, and gene function disruption can be performed with ease in these animals as compared to mice (89–92). Therefore, rather than demonstrating immunological events with select few lines, multiple lines of zebrafish can be generated and used (93, 94). Moreover, the zebrafish is an excellent model for tracing some infectious diseases such as tuberculosis. The granuloma formed by mycobacterial infection in zebrafish exhibits similar histological and pathological features as are evident in *Mtb* infected human granuloma lesions (95, 96). The zebrafish model could surely empower immunologists to visualize the cross regulation of Treg and Th17 cells in pathophysiology of diseases.

The functionality of Treg and Th17 cells can be measured by various *in vitro* assays and *in vivo* adoptive transfer approaches. *In vitro* functional assays include isolating and co-culturing Treg with identifiable non-Treg to measure the functionality (11, 97). Whether or not suppressive activity is contact dependent can be established using trans well assays (11). The responding cells used for suppressive assays can either be stimulated in a polyclonal manner or by antigen pulsed APCs (98).

## Comparison of Treg and Th17 Response in Humans, Rodents, and Non-Rodent Animals

Although there are considerable similarities in the function and phenotype of Treg as well as Th17 cells isolated from mice and humans, differences are also evident. Isoforms of Foxp3 that lack exon 2 or exon 7 exist in human, but not in mice suggesting that the differentiation pathways for Treg in humans and mice may differ (99). Stimulated CD4^+^CD25^−^ T cells in the presence of IL-2 and TGF-β, express Foxp3 and IL-2 acts to stabilize the expression (30). In the absence of TGF-β, Foxp3 could be expressed transiently in stimulated Foxp3^−^ T cells isolated from humans and to a lesser extent in mice but human cells express latent TGF-β on their surface (100–102). Reactive oxygen species are abundantly present during the initial stages of inflammation and can activate latent TGF-β to make it available for further differentiation into either Treg or Th17 cells. The stages of human Treg generation when TGF-β and IL-2 are critically involved are not yet clearly identified and most studies have concluded that these cytokines dominantly help stabilize Foxp3 expression (49). Varying degrees of epigenetic changes in the Foxp3 locus of human and mouse Treg have been observed (103, 104). Thus, the Foxp3 locus in humans is methylated to a greater extent as compared to that in mice suggesting human Treg take longer to adopt a phenotype similar to that of mouse Treg (30). The identification markers used for distinguishing human and mouse Treg also display discordance. Thus, even Foxp3 cannot be used for unambiguously defining Treg in humans, unlike in mice (105). Cells expressing sustained Foxp3 expression, however, are considered as suppressive cells. The recently described marker Nrp1 that distinguishes mouse tTreg from pTreg does not faithfully identify one subset or the other in humans (106).

IL-6 and TGF-β play a non-redundant role in the generation of mouse Th17, but this may not be true for human Th17 cell generation (107). IL-1β, TNF-α, and IL-23 are all effective inducers of ROR-γt in differentiating human Th17 cells (108). TGF-β may be dispensable for Th17 cell generation in humans but not in mice (101). The requirement of factors for differentiation of human Th17 cells, however, needs to be cautiously interpreted. Thus, most studies focusing on differentiation of human Th17 cells were performed using peripheral blood cells, and donors are expected to have an exposure to one or more antigens. Therefore, the starting population may not be naïve. Cells are more likely to be naïve when isolated from cord blood and for such cells to differentiate into Th17, TGF-β seems to be critically involved (107). Therefore, differentiation and transdifferentiation of human Th cells need to be fully understood for both naïve and committed cells in order to manipulate Th17 cell responses.

Regulatory T cells and to a lesser extent Th17 cells have been described to exist in most non-rodent animals as well. However, as is described for humans and mice, a mutation in Foxp3 and any subsequent phenotypic effect has not been described in other animals. This could be because of the rarity of such genetic disorders. Anti-human or mouse Foxp3 monoclonal antibodies that cross-react with xenogeneic Foxp3 molecule are used for immunophenotyping Treg in other animal species. Various domains of Foxp3 are conserved across different species and hence show appreciable cross reactivity (109). Foxp3 specific monoclonal antibodies were produced for some non-rodents such as cats and bovines to detect and measure Treg responses (110–112). In cats, an alternative splice variant of Foxp3 lacking exon 2 also exists, an observation similarly recorded for human Foxp3 (99). Surprisingly, when wild type and the variant lacking in exon 2 were expressed in a cell, the suppressive activity was enhanced, as compared a single version expressing cells suggesting a critical role of exon 2 in activity of Treg (111). Cytokines shown to promote Th17 responses in cats are IL-1β, IL-6, TGF-β, and IL-21 (113). Foxp3^+^ Treg have been demonstrated in animals that include pigs, cows, sheep, goat, horses, baboon, macaque, chimpanzee, harbor seals, and walrus (109). The Foxp3 expression could be induced in CD4^+^ as well as CD8^+^ T cells isolated from lymph node of healthy dogs that were stimulated with Con-A (114). A subset of cells that express Foxp3 at intermediate level, but not Foxp3 high cells, also expressed IFN-γ suggesting a plastic nature of such stimulated Treg as well as their tendency to acquire an effector phenotype (115). This could also means that those cells that express optimal level of Foxp3 are more stable as compared to those expressing it to a lower level and the latter cells are not fully committed to Treg phenotype. CD4^+^ T cells isolated from PBMCs could be efficiently polarized into Th17 cells using a poly-specific stimulator con-A and a combination of cytokines that include IL-6, IL-1β, and TGF-β (116). Foxp3^+^ Tregs in other species were also described. In fact, suppressor cells in domestic animals were described even before CD25^+^CD4^+^ T cells description in mice. In most of these studies, PBMCs stimulated with con-A for a few days acquired suppressive activity toward autologous and allogeneic blood cells (117), but phenotypic markers of these suppressor cells were not described. More recently, Foxp3 was detected not only in bovines αβ-T cells but also in a small proportion of γδ-T cells that were stimulated with Con-A (118, 119). In fact, a recent report suggested that in ruminants that includes bovines, γδ-T cells predominantly play a regulatory role by producing copious amounts of IL-10 and the contribution of CD4^+^CD25^+^ Foxp3^+^ T cells as regulatory cells is minimal (120). In small ruminants that include sheep and goats, Foxp3 expression was not only limited to CD4^+^ T cells, but was also detectable in other cells such as CD4^+^CD8^+^ T cells, CD4^-^CD8^+^ T cells, as well as double negative CD3^+^ T cells (109). The proportion of non-CD4^+^ T cells showing Foxp3 expression was variable however. The recorded variation in Treg responses could be attributed to a lack of appropriate reagents, pathophysiological condition of animals, and accessibility to tissues samples for analysis. Animals that are also used for meat purpose, the analyses could be performed using peripheral blood as well as accessing lymphoid organs from slaughtered animals.

In summary, Treg and Th17 cells are likely to be present in most vertebrate species as these cells are thought to have co-evolved (5). The contribution of Th17 cells and the cytokine IL-17 in the pathogenesis of some infectious diseases in some of the non-rodent animals has been described (116, 121). However, most of these studies are observational, and cells were isolated from peripheral blood samples only.

## Interconversion of Treg and Th17 Cells

Does plasticity of Th subsets confer any advantage to the host? The answer probably is in the affirmative. Thus, thymic regression with age limits T cell precursor frequency and the interconverting ability of different Th subsets could provide a facility for the generation of an appropriate helper T cell response required for an efficient adaptive immunity. The cytokines present in the milieu dictate the phenotype of cell upon differentiation, which is well appreciated (122). Functional alteration can include a loss of a useful function, gain of an undesirable activity, or a change in cell location from the site where they normally function. Naïve non-Treg (CD4^+^Foxp3^-^) are converted into Treg (CD4^+^Foxp3^+^) when stimulated in the presence of IL-2 and TGF-β (11). Similarly, the forced expression of Foxp3 converted conventional T cells into Treg that exhibited a suppressive activity (59). Treg may lose expression of Foxp3 but may not necessarily undergo functional changes (123). Alteration in a cell location is usually explained by differential expression of homing molecules and this relocation can also explain functional changes in some instances (40, 42). Relocation effects may help explain changes in Treg activity during different phases of an inflammatory response. In fact, during an acute inflammatory response, the number of Foxp3^+^ Treg in draining lymph node is reduced dramatically while their number increased in distal lymph nodes. This could mean that Treg prefer to stay in a non-inflammatory environment conceivably by modulating their homing receptors. This may also mean that Treg are more efficient in regulating responses that are milder in nature. Alternatively, those cells that reside in the most severe inflammatory environments and still retain the phenotype are more resilient and less likely to become non-Treg. All these issues have yet to be addressed adequately.

Among factors responsible for conferring stability and limiting, plasticity is the continuous availability of cytokines such as IL-2 (124). Treg that are deprived of IL-2 and potentially other cytokines are more inclined to change their phenotype (125). At a molecular level, this outcome can be explained in terms of epigenetic alterations in the conserved non-coding sequences (CNS) of *Foxp3* gene (126). Some have advocated that the subsets of Treg that are more plastic are those at an intermediate stage of their differentiation (122, 126). Such cells may eventually fail to establish their complete epigenetic architecture, an effect that can be influenced by the microenvironment (122). One of the most studied epigenetic modifications that is known to influence the stability of Treg is methylation of CpG islands in the CNS2 of *Foxp3* gene, also known as Treg-specific demethylated region (TSDR) (126). Thus, those Treg that have a hypomethylated Foxp3 TSDR are more stable as compared to those whose TSDR is hypermethylated (125, 126). This also relates to the expression of Foxp3 and its ability to promote expression of Treg associated genes. Accordingly, the activity of DNA methyl transferases in such cells may decide whether phenotypically stable cells will be generated or not. Nrp1 a molecules differentially expressed by tTreg is also involved in stabilization of Foxp3 expression. Signaling induced by ligation of Nrp1 with semaphorin-4a molecule in Treg-enhanced expression of transcription factors such as Foxo1 and Foxo3 to help stabilize Foxp3 expression (23). Eos is another transcription factor that impacts on the stability of Treg, but this effect could be independent of Foxp3 expression (127). Other studies indicate that Treg stability involves post translational modification of Foxp3 and the induction of its alternative splice variants (128). Treg that have enhanced phosphorylated Foxp3 (p-Foxp3) levels are more stable as compared to those that have less or no p-Foxp3 (128–130). Accordingly, phosphatases induced by a highly proinflammatory environment could dephosphorylate Foxp3 in Treg, which then are converted to become pathogenic Th17 cells (129, 130). Another study attributed the metabolic state of Th cells to their function and phenotype (131). Thus, it was shown that glycolysis in Th cells is critical for their conversion to become Treg (131). Enolase I, an enzyme, is induced when cell metabolism is switched to the glycolytic pathway (131). Enolase I plays an essential role by interacting with Foxp3 regulatory sequences to effect the expression of an alternative splice variant that utilizes exon 2 of Foxp3 (131). However, the mechanisms responsible for stability conferred by alternative splice variants of Foxp3 are not entirely clear, but could relate to their resistance to degradation or the presence of more amino acid residues that can undergo phosphorylation.

Some studies have implicated the role of certain microRNAs in regulating the stability of Treg (132–134). miRNAs are small oligonucleotides that are expressed endogenously and have critical roles in gene expression (132). In general, miRNA 29, 125a, 125b, 155, and 181 seem to affect differentiation of Th subsets (132). Some miRNAs such as miRNA 181 modulates TCR signaling and its expression alters with the maturation state of T cells (135). miRNA 155 specifically influences differentiation of Treg and Th17 cells which can affect the outcome of inflammatory diseases (136, 137).

As differentiation pathways between Treg and Th17 cells are shared, these cells exhibit greater tendency for interconversion. Some investigators have suggested that TGF-β induced Tregs as compared to natural Tregs are more likely to acquire a Th17 phenotype. Such cells are more likely to express membrane bound TGF-β and in an environment enriched in IL-6 or other inflammatory molecules, they become Th17 cells (138). Additionally, TGF-β induced cells have not established their complete epigenetic landscape and hence are more plastic in nature as compared to natural Treg. The conditions where Th17 cells can also become Foxp3 expressing Treg have not been established as yet, but the Th17 cells change to acquire other phenotypes that include Th1, Th2, Tr1, or T_FH_. This could occur because of the relative positioning of Foxp3 and ROR-γt in a 3-diamensional space in the cell and hence a physical interaction may not occur in Th17 cells as does occur in Treg (139). For establishing plasticity issues unambiguously, fate-mapping mice as described in an earlier section are used. Not only mice but also human Treg can become Th17 cells when stimulated with IL-1β and IL-6 (140). In conclusion, the interconversion of Foxp3^+^ Treg into Th17 cells is appreciable and well established upon the change of microenvironment but counterconversion of Th17 cells into Foxp3^+^ Treg cells is not known currently.

## Cross Regulation of Treg and Th17 Cells During Pathophysiology of Infectious Diseases

That Foxp3 is critically involved in the function of Treg has been shown in both humans and mice. A spontaneous mutation comprising a 2-bp insertion in the coding region of Foxp3 gene resulted in a truncated non-functional protein. Mutant mice, known as scurfy mice, developed spontaneous multiorgan inflammatory lesions (141–143). Male mice exhibited a pronounced phenotype as compared to females, suggesting the mutation was X-linked. Crossing scurfy mice with Foxp3 transgenics rescued the phenotype confirming the role of the mutation in disease causation (16). Similarly, patients who had immunodysregulation polyendocrinopathy enteropathy X-linked (IPEX) syndrome exhibited a mutation in the Foxp3 gene and developed autoimmune enteropathy, psoriasiform or eczematous dermatitis, nail dystrophy, and endocrinopathy. IPEX is a rare disease with a strong genetic association (144).

The balancing of response in activity of Treg and Th17 cells can influence the outcome of numerous infectious and non-infectious diseases (108). Whether or not these cells play a role in orchestrating disease due to infections in non-rodent animals is not well established and is suggested based on scanty data, which are often unconfirmed. During infections, the dominant effect of Treg perhaps is not to dampen protective immunity, but to prevent collateral tissue damage. In some infections, such as the one caused by *Leishmania*, Tregs that were induced *de novo* and recruited to infected sites were specific to pathogen-derived antigens (14, 145). Along similar lines, it was demonstrated that parasites (*Schistosome*) and bacteria (*Helicobacter pylori, Mycobacterium, Histoplasma)* promoted the peripheral generation of Treg (146–148). A protozoan parasite, *Toxoplasma gondii*, caused enhanced immunopathological reactions by inhibiting and destabilizing Treg (14, 145). Interestingly, destabilized Treg acquired Tbet and produced IFN-γ suggesting their conversion into Th1 like cells. In this study, ROR-γt expression by these was not analyzed. Tbet controls the expression of TIM-3 and those Tregs that express TIM-3 were shown to be resistant to apoptosis when ligated with galectin-9 (149). These seemingly contradicting observations could in fact hint the existence of different subtypes of Treg some of which will eventually be eliminated while some remain in animals and serve as dual function. How acute and chronic viral infections signal Treg response has been investigated (46). The outcome of acute infections caused by viruses such as Friend retrovirus, lymphocytic choriomeningitis virus (LCMV), influenza A virus (IAV), West Nile virus (WNV), respiratory syncytial virus (RSV), hepatitis A virus (HAV), and HSV-1 is influenced to a varying degree by Treg and possible Th17 cell responses (150). Acute LCMV infection induced type I interferon that diminished Treg function and as a result anti-viral CD4^+^ and CD8^+^ T cell responses are enhanced (151). Treg also critically influence the outcome of infection by IAV, WNV, RSV, HAV, and HSV-1 (69). In mice infected with IAV intranasally, more Foxp3^+^ Treg accumulate in the draining mediastinal LNs (MLNs) suggesting that virus is able to promote Treg responses (152). Mice depleted of Treg developed more severe lesion suggesting Tregs were able to control immunopathological responses. Respiratory influenza infection induced CCR9^+^CD4^+^ T helper cell generation in the MLNs. These cells, by responding to CCL25, preferentially migrated to the gut and were responsible for an inflammatory reaction mediated by Th17 cells (153). The antigen specificity and phenotype of migrating CD4^+^ T cells in the gut are not known. Whether or not the migratory cells by themselves orchestrated gut inflammation, or induced conversion of resident CD4^+^ T cells to become Th17 cells, remains to be elucidated. However, this study indicated that Th17 cells could in fact serve as one of the players of “common mucosal immune axis” and could influence the composition of microbiota in the gut during some infections (153). Another study demonstrated that influenza virus inhibited Th17 mediated control of a secondary bacterial infection to cause pneumonia (154). Therefore, during IAV pathogenesis, the cross play of Treg and Th17 can impact the pathogenesis. During WNV infection in humans, Treg helped control the development of clinical symptoms and fever by preventing tissue damaging inflammatory reactions because asymptomatic individuals had greater numbers of Treg in peripheral blood (155). Similarly, WNV infected mice, depleted of Treg, developed lethal encephalitis suggesting Treg response was protective in nature (155). A specific role of Th17 cells in WNV pathogenesis has not been demonstrated, but encephalitis caused by WNV was not influenced by the Th17 cell response (156). As compared to controls, Treg depleted mice upon RSV infection showed enhanced Th2 responses that led to severe pulmonary immunopathological lesions (157). Most cases of acute HAV infection resolve with efficient viral clearance and innocuous pathological consequences, which could relate to how Treg are signaled (158). HAV directly binds to its cellular receptor 1 (HAVCR1 also known as TIM-1) expressed by Treg and as a result abrogates their function to promote anti-viral CD8^+^ T cell responses. Efficient CD8^+^ T cells then help control virus infection (159). Whether or not Th17 cells play any role in RSV and HAV infection is not clear. The influence of Treg in HSV pathogenesis has been extensively studied by numerous approaches (160–164). Mice that were depleted of Treg prior to HSV infection mounted enhanced primary and memory anti-viral CD8^+^ T cell responses (162) and when Treg were depleted prior to ocular infection with HSV-1 heightened CD4^+^ T cell effector response led to an aggravated corneal inflammatory disease, as compared to those mice that had intact Treg responses (163). This observation was followed up in subsequent studies employing adoptive transfer of natural Treg as well as TGF-β induced Treg in mice before infection (161, 163). Treg recipient mice developed diminished inflammatory lesion as compared to infected controls (161). We observed that ligation of CD4^+^ T cell expressed sphingosine 1 phosphate receptor (S1P1) by an agonist FTY720 promoted Treg responses (165, 166). These converted cells, however, were inclined to acquire a Th17 phenotype when incubated with IL-6 and exhibited an aggressive proinflammatory activity in HSV-1 infected animals (165). IL-6 neutralization diminished lesions of the disease suggesting that the converted cells might be more plastic and in fact more damaging. What stage of infection Treg responses are critical in controlling the disease severity was investigated using a DTR-Foxp3 transgenic mouse model in which Treg could be depleted using diphtheria toxin at different times post-infection (70). The results suggested that Tregs continue to regulate inflammatory responses irrespective of stage when these are depleted and that Treg might in fact be acting both in the DLN during induction phase of response and at inflammatory sites (70). Direct interaction of Treg expressed HVEM and HSV-1-gD glycoprotein provided a partial explanation as to how HSV-1 is able to signal Treg so promptly after infection (167). The role of Th17 cells in HSV-1 induced pathogenesis was also investigated using IL-17R KO mice as well as in mice lacking different subunits of cytokine IL-23 (p19 and p35), a cytokine critically involved in promoting Th17 cell responses (168, 169). These studies demonstrated that IL-17 contributed by innate immune cells, γδ T cells and Th cells, enhanced the severity of inflammatory lesions. Th17 cells were predominantly involved during the chronic phase of infection, while during the acute phase their contribution was minimal (169). This also suggests that inflammatory milieu in cornea may induce conversion of some accumulated Treg or Th1 cells into a Th17 phenotype. It would be worth investigating whether Th17 cells can further become Treg and how would that influence the lesion severity.

Most chronic viral infections were shown to influence Treg responses and eventually the outcome of chronic infections (13, 46). Notably, HIV and HCV are the most prominent chronic viral infections where Treg seems to play a critical role in pathogenesis (13, 46). Precise mechanisms how these infections trigger Treg responses are not clear, but the microenvironments created could contribute. HIV, HCV, and IAV could all activate latent TGF-β to promote Treg and potentially Th17 responses depending on its concentration along with that of other inflammatory cytokines (43, 170, 171). During HIV infection, Tregs play multiple roles that range from an early abrogation of effector CD4^+^ T cells to tissue repair during later stages (172). HIV promotes Treg responses by modulating the function of DCs which stimulate Treg generation (173). Tregs, in turn, control the activation of CD4^+^ T cells to minimize their infection by the virus. Thus, activated CD4^+^ T cells are more susceptible to HIV infection as compared to those in resting stage (174). TGF-β produced by Treg, and probably other cells, promotes collagen deposition in lymphoid organs (175). This poses a problem when the patients are given anti-retroviral therapy and immune reconstitution is required. Thus, the effective space available would be less for immune reconstitution (175). The involvement of Treg in HIV pathogenesis, therefore, is a complex issue and needs more study.

Th17 cells seem to play a crucial role in the pathogenesis of HIV infection as these cells accumulate abundantly in the gut-associated lymphoid tissues (GALT) early after infection (176). Whether the accumulated Th17 cells in GALT originate from Treg or differentiate from naïve cells is still to be established. The activated Treg in the gut could contribute to TGF-β production and HIV infection could trigger IL-6 production by innate cells. Th17 cells are known to express surface CD45RO, CCR5, and CXCR4 making them more permissive to HIV infection (177). Infected Th17 cells are cleared by the virus itself, or by cytotoxic CD8^+^ T cells. As Th17 cells are critical for maintaining the integrity of mucosal barriers, their depletion could disrupt these barriers and initiate generalized immune activation (178, 179). The Th17 cells that influence the outcome of HIV infection may not necessarily be specific for viral antigens. The role of Th17 cells was also demonstrated in long-term non-progressers who exhibit pronounced Th17 responses as compared to those who progress rapidly to develop HIV-AIDS. Restoration of Th17 cells in patients undergoing highly active anti-retroviral therapy is an indicator of better prognosis predominantly due to efficient control of bacterial infections by these cells (180). Therefore, a balance of Treg and Th17 cell response may critically influence the pathogenesis of HIV infection.

HCV and Treg interaction is complicated to investigate, as the responses need to be evaluated in the liver, where disease occurs. This is particularly confounded by the unavailability of a rodent model and the now unavailable chimpanzee being the only reliable animal model to study HCV pathogenesis. What determines the resolution of infection in only 20% HCV infected patients is not clearly understood but is thought to be explained by an effective anti-viral CD8^+^ and CD4^+^ T cell response (181, 182). In those which fail to control infection, some have advocated that an induced Treg response, which blunts the activity of effector T cells, could be the explanation (183). During HCV infection, the cell types that are known to exhibit predominant regulatory activity are Tr1 cells and possibly CD8^+^ Treg in addition to Foxp3 positive cells as suggested by some studies (170). However, it remains to be evaluated whether Tregs play a beneficial or detrimental role during chronic stages of HCV infection. Th17 cells, owing to their cytokine secretion, are thought to play a predominant role in the repair process leading to fibrosis in the liver and seem not to play a critical role early during HCV infection. Accordingly, patients treated with interferon and ribavirin therapy had decreased Treg responses but minimal effects on Th17 cells were observed (183). The Treg and Th17 cell ratio, however, was skewed toward Th17 cells with a favorable outcome of therapy. How various subsets of Th cells influence HCV pathogenesis remains a controversial issue that merits further evaluation. However, the issue is now less relevant since there is a new highly effective anti-viral that controls HCV infection.

Regulatory T cells, and to a lesser extent Th17 cells, do influence the outcome of various infections in pet animals that include dogs and cats. These animals also serve as models for various infectious and non-infectious diseases. For example, similarities in the pathogenesis of feline immunodeficiency virus (FIV) and HIV make the cat a useful animal model (184). FIV was shown to infect Treg and this made them better suppressors (113, 185). FIV infected cats exhibit an early depletion of CD4^+^ T cells and enhanced Treg activity, which in turn compromises anti-viral adaptive immunity. This provides the virus an opportunity to establish a productive infection (186). More recent reports suggest a dysregulation of Treg and Th17 cells during FIV pathogenesis in cats during a systemic infection as well as in the placenta leading to non-viable pregnancies (113). Whether or not a similar situation exists in pregnant women infected with HIV is not known.

The canines genome revealed striking similarities in functionally related genes with humans and single nucleotide polymorphisms have been recently mapped (187). Some shared infections between dogs and humans are beginning to provide new insights in the pathophysiology of diseases (188). Foxp3^+^ Treg responses have been studied in canine leishmania infection where a variable response pattern for Treg and Th17 cells was observed in different organs (189). Whether or not interconversion in these cell populations occurs during infection is yet to be explained.

The responsiveness of Treg during infectious diseases in bovines has been investigated (190–192). *Mycobacterium paratuberculosis*, the causative agent of debilitating Johne’s disease and bovine leukemia virus (BLV) induce CD4^+^ T cells that produced IL-10 and those that expressed Foxp3, respectively (190, 192). During BLV infections, enhanced Treg responses act to constrain anti-viral immunity and probably cause the pathogen to persist in animals (191). Johne’s disease is thought to be orchestrated by Th1 cells of which some cells also produced IL-17 suggesting the plastic nature of these cells. However, as this is mainly a gut associated disease, probably the role of balance between Tregs and Th17 cells would provide better insights into its pathogenesis.

Small ruminants, such as sheep and goats, serve as major livestock for landless laborers and marginal farmers. Which cellular mediators are induced early during the response decides the efficiency of immunity to infections as well as immunization. Major pathogens that infect small ruminants are parasites such as *Teladorsagia circumcincta* and *Haemonchus contortus*, which induce an orchestrated response pattern characterized initially by Th1 and during later stages by Th2 and regulatory response (193, 194). Whether or not Th17 responses are critical for defense against parasitic infections has not been investigated. As these parasites infest gut of these animals, it would be interesting to investigate how a balance of Th17 and Treg is affected. Rinderpest virus is the only pathogen of animals eliminated from the face of earth; however, its close relative pestes des petits ruminantium virus (PPRV) is still a major problem in many parts of the world in ruminants and cause immunosuppression in the host. Both viruses inhibit proliferation of leukocyte *in vitro* (195). Surprisingly, however, the role of Treg and Th17 during PPRV infection or during vaccination against PPRV has not been investigated and could provide better insights into their pathogenesis and eventually better management practices could be employed.

## Conclusion

Enumerable studies performed in rodents and to some extent in humans exposed to or infected with one or more microbes revel an intricate interplay of various subsets of CD4^+^ T cells which influences the disease outcome. Treg and Th17 response dynamics is beginning to provide new insights into the pathogenesis of various infections. However, there exist a vast gap in our understanding how these cell type are induced, maintained, and interact with each other in animals other than inbred rodents. Such insights could open new avenues of modifying their function to achieve better resolution of infection and mitigate tissue damaging reaction in humans and animals.

## Author Contributions

SS compiled information and discussed in context, while BR was involved in editing and logically presenting the information.

## Conflict of Interest Statement

The authors declare that the research was conducted in the absence of any commercial or financial relationships that could be construed as a potential conflict of interest.
